# Abnormal Gray Matter Volume and Functional Connectivity in Parkinson's Disease with Rapid Eye Movement Sleep Behavior Disorder

**DOI:** 10.1155/2021/8851027

**Published:** 2021-02-22

**Authors:** Xu Jiang, Zhuang Wu, Min Zhong, Bo Shen, Jun Zhu, Yang Pan, Jun Yan, Wenbin Zhang, Pingyi Xu, Chaoyong Xiao, Li Zhang

**Affiliations:** ^1^Department of Geriatrics, Nanjing Brain Hospital Affiliated to Nanjing Medical University, Nanjing, China; ^2^Department of Neurosurgery, Nanjing Brain Hospital Affiliated to Nanjing Medical University, Nanjing, China; ^3^Department of Neurology, The First Affiliated Hospital of Guangzhou Medical University, Guangzhou, China; ^4^Department of Radiology, Nanjing Brain Hospital Affiliated to Nanjing Medical University, Nanjing, China

## Abstract

**Objective:**

Rapid eye movement (REM) sleep behavior disorder (RBD) is a common symptom in Parkinson's disease (PD), and patients with PD-RBD tend to have an increased risk of cognitive decline and have the tendency to be akinetic/rigidity predominant. At the same time, the mechanisms of RBD in patients with PD remain unclear. Therefore, this study aimed to detect the structural and functional differences in patients with PD-RBD and PD without RBD (PD-nRBD).

**Methods:**

Twenty-four polysomnography-confirmed patients with PD-RBD, 26 patients with PD-nRBD, and 26 healthy controls were enrolled. Structural and functional patterns were analyzed based on voxel-based morphometry and seed-based functional connectivity (FC). Correlations between altered gray matter volume (GMV)/FC values and cognitive scores and motor impairment scores in PD subgroups were assessed.

**Results:**

Compared with patients with PD-nRBD, patients with PD-RBD showed relatively high GMV in the cerebellar vermis IV/V and low GMV in the right superior occipital gyrus (SOG). For the FC, patients with PD-RBD displayed lower FC between the right SOG and the posterior regions (left fusiform gyrus, left calcarine sulcus, and left superior parietal gyrus) compared with the patients with PD-nRBD. The GMV values in the right SOG were negatively correlated with the Unified PD Rating Scale-III scores in patients with PD-RBD but positively correlated with delayed memory scores. The GMV values in the cerebellar vermis IV/V were positively correlated with the tonic chin EMG density scores. There were positive correlations between the FC values in the right SOG-left superior parietal gyrus and MoCA and visuospatial skills/executive function scores and in the right SOG-left calcarine sulcus and delayed memory scores.

**Conclusion:**

Higher GMV in the cerebellum may be linked with the abnormal motor behaviors during REM sleep in patients with PD-RBD, and lower GMV and FC in the posterior regions may indicate that PD-RBD correspond to more serious neurodegeneration, especially the visuospatial–executive function impairment and delayed memory impairment. These findings provided new insights to learn more about the complicated characteristics in patients with PD-RBD.

## 1. Introduction

Rapid eye movement sleep behavior disorder (RBD), which is characterized by nightmares, abnormal motor manifestations, and impaired muscle atonia during REM sleep [1], is closely associated with Parkinson's disease (PD). RBD can occur not only in the prodromal stage of PD but also in the course of PD [2]. The prevalence of RBD in PD can range from 35% to 60% [3]. RBD is one of the strongest markers of the diffuse/malignant PD phenotype [4], and patients with PD-RBD are reported to have an increased risk of cognitive impairment, such as visuospatial–executive function and motor alternations, for example, tending to be akinetic/rigidity (AR) predominant [5, 6]. However, little is known about the potential causes of this condition.

The mechanisms of RBD symptoms are still controversial. Previous studies have proven that the impaired subcoeruleus nucleus and ventral medial medulla are associated with generating abnormal motor behaviors during REM sleep [7, 8]. However, as research into this subject deepens, investigators have found that the impaired brainstem structures may not be sufficient to cause RBD symptoms and are currently paying closer attention to the neocortex and limbic system. Iranzo et al. [8] proposed two hypotheses: one was the cortical hypothesis and the other was the brainstem hypothesis. In normal REM sleep, the ventral medial medulla can inhibit the spinal cord and prevent the motor cortex from generating movements owing to hyperpolarization of the spinal cord. Therefore, the cortical hypothesis proposed that the neocortex can produce movements because of the impaired ventral medial medulla in RBD. Similarly, during normal REM sleep, the ventral medial medulla also can inhibit the red nucleus and prohibit the spinal cord from producing movements. Thus, the brainstem hypothesis stated that the red nucleus can generate excessive movements on account of the impaired ventral medial medulla in RBD. None of the two hypotheses involved the limbic system. However, Guo et al. [9] found that increased nodal measures in the neocortex and limbic system may stimulate the ascending reticular activating system, resulting in a “like-arousal” state during REM sleep and generating abnormal motor behaviors in patients with PD-RBD. In contrast, Li et al. [10] showed that decreased activity of the primary motor cortex may lead to poor control of motor behaviors during REM sleep in patients with PD-RBD. These inconsistent results and different explanations urge us to learn more about the complex mechanisms underlying the RBD symptoms in PD.

Voxel-based morphometry (VBM), which is an effective method of analysis of the change in gray matter volume (GMV) at the voxel level [11], has been used to explore the structural alternations separately in patients with PD-RBD. Some studies have found that these patients had reduced gray volume in the thalamus, putamen, lingual gyrus, cerebellum, pontomesencephalic tegmentum, amygdala, anterior cingulate gyrus, posterior cingulate gyrus, and hippocampus [12–16]. However, some methodological limitations are still shown in these studies, especially the lack of diagnosis of RBD by polysomnography (PSG) [13, 15]. Studies have indicated changes in functional connectivity (FC) in idiopathic RBD [17, 18], but research on FC in PD-RBD is scarce, and no study to date has explored the structural and functional differences between PD-RBD and PD without RBD (PD-nRBD) in combination with VBM and FC. The GMV has been seen as an important driving factor for the changes of FC, and the coupling of GMV and FC may be more liable to discover and identify the relevant functional components than the single one [19, 20]. Simultaneously, the combination of VBM and FC analysis has been used in different diseases to further explore the complex substrate [21–23]. Therefore, VBM and FC analysis in PD patients with PSG-confirmed RBD, PD-nRBD, and healthy controls (HCs) were used to investigate the structural and functional alternations, and correlation analysis was used to explore whether the altered covariances were associated with the chin EMG activity, motor symptoms, and cognition. We postulated that PD-RBD patients would show more extensive and severe cortical alternations in terms of structure and function, which may be in relation to the chin EMG activity, motor symptoms, and cognition.

## 2. Materials and Methods

### 2.1. Participants and Clinical Evaluations

Fifty patients with PD, including 24 polysomnography-confirmed RBD cases and 26 HCs who were matched to patients with PD in terms of age, education, and sex, were recruited from the Nanjing Brain Hospital Affiliated to Nanjing Medical University from July 2015 to September 2019. The inclusion criteria were (1) a diagnosis of PD patients established by movement-disorder specialists in accordance with the UK Brain Bank PD criteria [24]; (2) age from 51 to 74 years; (3) PD duration ≤12 years; and (4) Hoehn & Yahr (H&Y) stage ranging from 1 to 4; and (5) PD patients who were right-handed. The exclusion criteria were (1) parkinsonism other than PD; (2) dementia; (3) psychiatric disorder including relevant depression, anxiety, and schizophrenia according to DSM-5 criteria [25]; (4) obstructive sleep apnea syndrome with an apnea-hypopnea index >5; (5) history of stroke, brain tumor, drug abuse, cardiovascular disease, metabolic disease, and chronic obstructive pulmonary disease; and (6) history of epilepsy. No subjects were excluded, and all participants signed informed written consent and provided clinical and imaging data.

All clinical information was evaluated. The motor symptoms and severity were measured using the Unified PD Rating Scale-III (UPDRS-III), H&Y stage, levodopa equivalent daily dose, and AR scores evaluated by items 22–27 and 31 of UPDRS-III [26]. The global cognitive function and mental status were assessed using the Montreal Cognitive Assessment (MoCA) and the Hamilton Depression Rating Scale (HAMD).

### 2.2. PSG

All subjects were evaluated using the RBD Sleep Questionnaire (RBDSQ), and 24 patients who met the criteria of a score ≥5 were considered as possible patients with PD-RBD [27], while the remaining subjects were recruited as PD-nRBD and HCs. Considering that the sensitivity and specificity of the RBDSQ are 0.842 and 0.962 [28], respectively, we conducted the one-night PSG to confirm further the patients with RBD among the possible PD-RBD cases. According to the diagnostic criteria of RBD of ICSD-3, patients who were lack of muscle atonia during REM sleep and who exhibited aberrant motor behaviors documented by PSG or based on clinical history were classified as RBD [1]. All the 24 possible patients with PD-RBD were eventually confirmed as the definite patients with PD-RBD.

Possible patients with PD-RBD underwent one-night video-PSG (more than 8 h generally) at the sleep center of the Nanjing Brain Hospital Affiliated to Nanjing Medical University. The PSG recording consisted of electroencephalogram (six standard electrode derivations, including C3-A2, C4-A1, O1-A2, O2-A1, F3-A2, and F4-A1); bilateral electrooculograms; chin electromyogram (EMG); electrocardiogram; snoring, oral, and nasal airflow; thoracic and abdominal movements; body position; bilateral limb electromyograms; and pulse oxygen saturation. The results of PSG were assessed by professional technicians and the sleep stages were scored relying on the criteria drafted by The American Academy of Sleep Medicine [29]. The REM sleep was scored in accordance with the following method: the first REM occurred as the onset of REM sleep, and the REM sleep terminated when a specific brainwave of other sleep stages, such as K complex and sleep spindle or awakening EMG signal, emerged or rapid eye movements were absent for 3 min [30]. The tonic chin EMG activity and phasic chin EMG activity during the REM phase were identified and quantified in light of the standard established by Montplaisir et al. [30]. A tonic chin EMG activity >30% of the total REM sleep time or a phasic chin EMG activity >15% of the total REM sleep time was considered as aberrant muscle atonia during REM sleep [30].

### 2.3. Magnetic Resonance Imaging (MRI) Data Acquisition

A 3.0-Tesla MRI scanner (Siemens, Germany) was used to collect the MRI data of all subjects and the patients with PD underwent MRI scanning in the off state. The participants were instructed to lie down, relax, keep their eyes closed, and stay awake during scanning. Their heads were fixed using sponge mats to control head movement, and earplugs were inserted in the ears to reduce noise. High-resolution T1-weighted images were acquired using the 3D magnetization-prepared rapid gradient-echo (3D-MPRAGE) sequence with the following conditions: repetition time (TR) = 1900 ms, echo time (TE) = 2.48 ms, flip angle (FA) = 9^o^, matrix size = 256 × 256, field of view (FoV) = 250 × 250 mm, slice number = 176, slice thickness = 1 mm, and slice gap = 0 mm. Resting-state functional images were acquired using an echo-planar imaging sequence, with TR = 2000 ms, TE = 25 ms, FA = 90^o^, matrix size = 64 × 64, FOV = 240 × 240 mm, slice number = 33, slice thickness = 4 mm, and slice gap = 0 mm.

### 2.4. Gray Matter Volume (GMV) Analysis

Structural image processing was performed using the Statistical Parametric Mapping software (SPM8; http://www.fil.ion.ucl.ac.uk/spm) in MATLAB 2013b (MathWorks, Natick, MA). First, we implemented VBM to segment the structural images into gray matter (GM), white matter, and cerebrospinal fluid. The segmented GM and white matter images were then normalized to the standard Montreal Neurological Institute template with nonlinear modulation. Finally, the normalized images were smoothed using the 8 mm full-width at half-maximum isotropic Gaussian kernel.

### 2.5. Resting-State Functional Image Preprocessing

**Figure 1:**
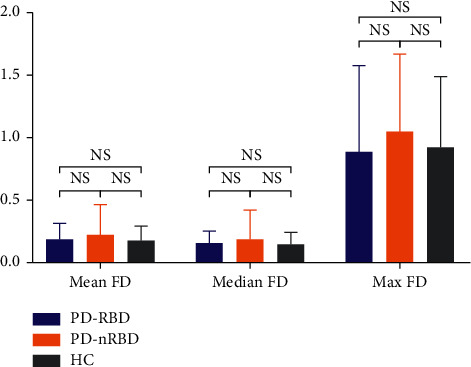
The comparison of framewise displacement between the groups. “NS” means no significance.

The preprocessing of fMRI images was performed using Data Processing Assistant for Resting-State fMRI (DPABI) [31]. The first 10 volumes of fMRI images were discarded to achieve magnetization equilibrium effects, and slice timing and realigning procedures were used to correct the time differences and head movement effects. No subjects were excluded based on head motions greater than 2.5 mm of translation or 2.5° of rotation. Considering motion-associated differences among the subjects, individual mean framewise displacement (FD), median FD, and max FD were calculated based on the formula of a previous study [32]. No significant differences were shown for mean FD, median FD, and max FD between groups (Figure 1). The remaining images were spatially normalized using the echo-planar imaging template, resampled to 3.0 × 3.0 × 3.0 mm^3^ voxel size, and smoothed with a Gaussian kernel of 4 × 4 × 4 mm. Then, the white matter signal, cerebrospinal fluid signal, global mean signal, and 24 head motion parameters were regressed. The resulting data were filtered (0.01–0.1 Hz) to reduce low-frequency drift and cardiac noise. Finally, scrubbing was performed.

### 2.6. FC Analysis

Regions exhibiting significant differences in GMV between patients with PD-RBD and PD-nRBD were considered as regions of interest for FC analysis. The mean time series of the regions of interest were extracted for each participant, and the FC maps were obtained by calculating the temporal correlation coefficients between the mean time series of seed regions and the time series of each voxel within the whole brain. The normalized FC maps were then created by Fisher's Z transformation.

### 2.7. Statistical Analysis

#### 2.7.1. Clinical Data

Age, MoCA, HAMD, RBDSQ scores, tonic chin EMG density scores, and phasic chin EMG density scores were analyzed using the one-way analysis of variance (ANOVA) among patients with PD-RBD, PD-nRBD, and HCs. Because of the nonnormal distribution of the data of education, MoCA domain scores were analyzed using the Kruskal–Wallis test among the three groups. The Chi-squared test was used to study the data of sex among the three groups. Two-sample *t*-tests were applied in the analysis of disease duration, UPDRS-III, AR scores, H&Y stage, and levodopa equivalent daily dose between the PD-RBD and PD-nRBD groups. These statistical analyses were performed using the SPSS 23.0 software (Chicago, IL), with the significance level set at *P* < 0.05.

#### 2.7.2. GMV and FC Data

GMV and FC differences were identified via the one-way ANOVA among the three groups using age, gender, education, and total intracranial volume as covariates in the REST software. The significance level of GMV was set at cluster-level corrected *P* < 0.05 with AlphaSim correction with voxel-level *P* value <0.001. Then, the maps of GMV and FC differences among the three groups were extracted as the masks. The mean GMV and FC values of each cluster were extracted in each subject based on the masks. The post hoc *t*-test was conducted in each cluster between the groups using the SPSS 23.0 statistical analysis software. Multiple comparison correction was performed with the Bonferroni correction, where *P* < 0.017 was considered significant. Then, Pearson's correlation analysis between the GMV/FC values and the MoCA, UPDRS-III, tonic chin EMG activity and phasic chin EMG activity, and Spearman's correlation analysis between the GMV/FC values and the MoCA domain scores were evaluated in the PD-RBD and PD-nRBD groups using SPSS 23.0 with a significance threshold of *P* < 0.05. Bonferroni correction was used for multiple comparisons in the correlation analysis.

## 3. Results

### 3.1. Demographic and Clinical Data

As presented in Table 1, no significant differences were detected among the three groups in terms of age, education, sex, MoCA, naming, attention, language, abstraction, orientation, and HAMD. Moreover, disease duration, UPDRS-III, AR scores, H&Y stage, and levodopa equivalent daily dose were not significantly different between the PD-RBD and PD-nRBD groups. In addition, patients with PD-RBD showed lower delayed memory scores than did those with HCs. Finally, patients with PD-RBD achieved higher RBDSQ scores, tonic chin EMG density scores, and phasic chin EMG density scores and lower visuospatial skills/executive function scores than did those with PD-nRBD and HCs.

### 3.2. GMV Data

**Figure 2:**
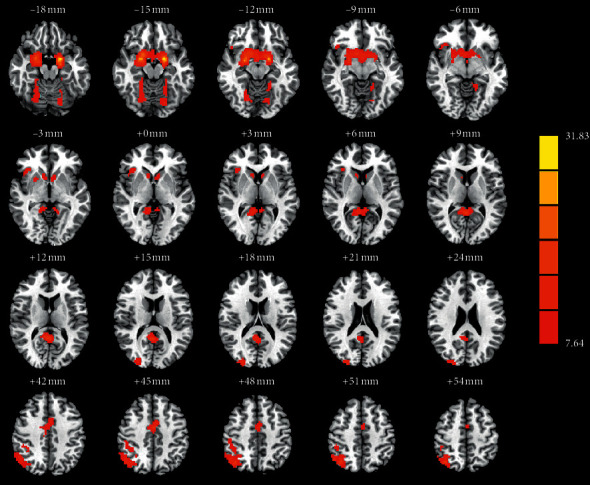
The significant differences of gray matter volume (GMV) and functional connectivity (FC) among patients with PD-RBD, PD-nRBD, and HCs. The results were displayed in MNI space, and red color represents the different brain regions.

**Figure 3:**
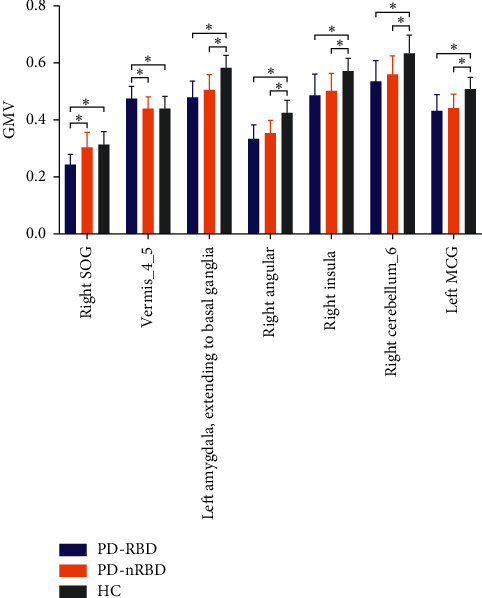
The significant differences of gray matter volume (GMV) between the groups. The results were indicated with (^*∗*^), ^*∗*^*P* < 0.017.

As shown in Figure 2 and Table 2, a one-way ANOVA exhibited significant group differences in GMV among the PD-RBD, PD-nRBD, and HCs groups in the right superior occipital gyrus (SOG), cerebellum (cerebellar vermis IV/V and right cerebellum lobule VI), limbic system (left amygdala and left middle cingulate gyrus (MCG)), basal ganglia (bilateral caudate nucleus (CN) and bilateral putamen), and right angular and right insula. The post hoc analysis revealed that the PD-RBD group had higher GMV in the cerebellar vermis IV/V and lower GMV in the right SOG compared with the PD-nRBD group. Patients with PD-RBD displayed lower GMV in the right SOG, limbic system (left amygdala and left middle cingulate gyrus), basal ganglia (bilateral caudate nucleus (CN) and bilateral putamen), right angular, right insula, and right cerebellum lobule VI compared with the HCs. Compared with the HCs group, the PD-nRBD group had relatively low GMV in the limbic system (left amygdala and left middle cingulate gyrus), basal ganglia (bilateral caudate nucleus (CN) and bilateral putamen), right angular, right insula, and right cerebellum lobule VI (Figure 3).

### 3.3. FC Data

We further explored the changes in FC among the PD-RBD, PD-nRBD, and HC groups based on the significant differences observed between the PD-RBD and PD-nRBD groups in the cerebellar vermis IV/V and right SOG.

**Figure 4:**
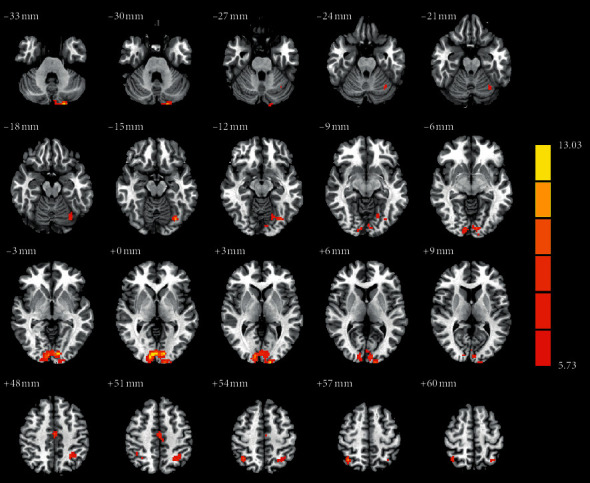
The significant differences of functional connectivity (FC) among patients with PD-RBD, PD-nRBD, and HCs. The results were displayed in MNI space, and red color represents the different brain regions.

**Figure 5:**
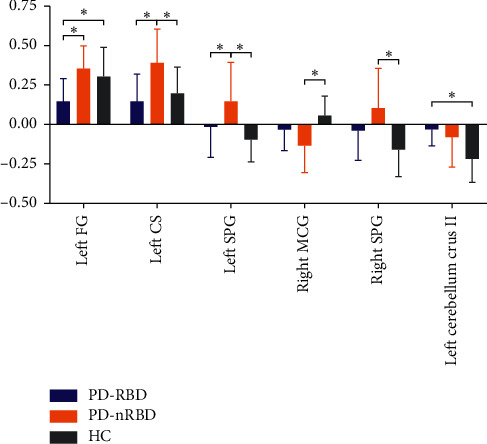
The significant differences of functional connectivity (FC) between right superior occipital gyrus (SOG) and other brain regions between the groups. The results were indicated with (^*∗*^), ^*∗*^*P* < 0.017.

As illustrated in Figure 4 and Table 2, a one-way ANOVA of FC analysis revealed the connectivity between the right SOG and the posterior regions (the left fusiform gyrus (FG), left calcarine sulcus (CS), left superior parietal gyrus (SPG), and right SPG), right MCG, and left cerebellum crus II. Regarding the FC of the right SOG, the post hoc analysis revealed that the PD-RBD group had a lower FC in the posterior regions (left FG, left CS, and left SPG) compared with the PD-nRBD group. Compared with the HCs group, the PD-RBD group showed a higher FC in the left cerebellum crus II and lower FC in the left FG. Compared with the HCs group, a relatively higher FC in the posterior regions (left CS, bilateral SPG) and relatively lower FC in the right MCG were found in the PD-nRBD group (Figure 5). However, the FC between the cerebellar vermis IV/V and other regions of the whole brain was not significantly different among the three groups.

### 3.4. Correlation Analysis

In the PD-RBD group, the GMV values in the right SOG were negatively correlated with the UPDRS-III scores (*r* = −0.44, *P*=0.03), but positively correlated with the language scores (*r* = 0.45, *P*=0.03) and delayed memory scores (*r* = 0.50, *P*=0.01); the GMV values in the cerebellar vermis IV/V were positively correlated with the tonic chin EMG density scores (*r* = 0.54, *P*=0.01). There were positive correlations between the FC values in the right SOG-left SPG and MoCA (*r* = 0.52, *P*=0.01) and visuospatial skills/executive function scores (*r* = 0.66, *P*=0.00) and in the right SOG- left CS and delayed memory scores (*r* = 0.41, *P*=0.04). In the PD-nRBD group, the GMV values in the cerebellar vermis IV/V were positively correlated with the UPDRS-III scores (*r* = 0.42, *P*=0.04). There were negative correlations between the FC values in the right SOG-left cerebellum crus II and the attention scores (*r* = −0.47, *P*=0.02) and the UPDRS-III scores (*r* = −0.46, *P*=0.02) (Tables 3 and 4).

## 4. Discussion

In this study, we applied the VBM and FC analysis to identify the meaningful structural and functional changes between patients with PD-RBD and those with PD-nRBD. First, higher GMV was observed in the cerebellum in the PD-RBD group versus the PD-nRBD group. The GMV values in the cerebellum were positively correlated with the tonic chin EMG density. Second, patients with PD-RBD exhibited the lower GMV in the occipital cortex (such as the right SOG) and the lower FC in the right SOG and the posterior regions than did those with PD-nRBD. In the PD-RBD group, the GMV values in the right SOG were negatively correlated with the UPDRS-III scores, and the GMV values in the right SOG and posterior regions were positively correlated with the MoCA scores and MoCA domain scores. Although the PD-RBD and PD-nRBD groups showed no significant differences in the basal ganglia, the lower GMV was detected in this structure (CN and putamen) between the PD-RBD/PD-nRBD group and the HCs group, suggesting that the structural abnormality of the basal ganglia is associated with PD. These findings may strengthen our understanding of the RBD symptoms in PD.

The cerebellum is involved in the generation of atonia during the REM phase [33]. Moreover, it was hypothesized that the cerebellum can receive the excitatory and inhibitory projection from the amygdala, thalamus, and vestibular nucleus and outputs to the brainstem during the REM phase [34]. Han and colleagues reported a study of patients with idiopathic RBD showing a positive correlation between the phasic chin EMG activity and the GMV in the cerebellum [35], and the presence of increased GMV in the cerebellar vermis IV/V patients with PD-RBD and positive correlation between the GMV values and the tonic chin EMG density was also observed in our study. These findings may clarify that the impaired cerebellum may be linked with the abnormal motor behaviors during REM sleep in these patients by affecting their projection to the brainstem structures. However, we did not find a significant FC difference in the cerebellar vermis IV/V between the PD-RBD and PD-nRBD groups, possibly due to the fact that the number of participants in this study may be insufficient to explore the FC differences in the cerebellar vermis IV/V.

Pathological studies have demonstrated that, with the Lew body disorders developing, the *α*-synuclein deposits are observed initially in the olfactory bulb (stage I), followed by their propagation to the brainstem (stage IIa), limbic system (stage IIb), brainstem and limbic system (stage III), and neocortex (stage IV), sequentially [36], and the misfolded *α*-synuclein may spread by the way of prion-likeness [37]. In our study, the two PD subgroups showed structural abnormalities in the limbic system (such as the amygdala) and neocortex (such as the angular). However, the PD-RBD group had relatively extensive structural abnormalities, such as the SOG, which was similar to the result of a previous study [38]. Simultaneously, both PD subgroups showed the structural alternations in the basal ganglia, which may concur with the hypothesis that the pathology from the basal ganglia may spread to the cortex through the corticopetal systems and result in the cortical thinning in the patients with PD-RBD and PD-nRBD [14]. Occipital gyrus and parietal gyrus, as part of the posterior regions, have been broadly confirmed to play an important role in visuospatial–executive function [39, 40]. Decreased brain activity in the occipital lobe has also been reported to be associated with the delayed memory impairment [41]. A previous study has found that patients with idiopathic RBD showed decreased GMV in the occipital and parietal lobe, which were correlated with the visuospatial loss [42]. These results were similar to our study that the PD-RBD group showed reduced GMV and FC in the posterior regions than did those with PD-nRBD and significant correlation with the cognitive and motor impairment, indicating that the PD-RBD may correspond to more serious neurodegeneration and exhibit predictive value regarding the progression of motor symptoms and cognitive function, especially the visuospatial–executive function and delayed memory [43].

The limitations of our study should be noted. First, the relatively small sample size may have hampered the analysis of the structural and functional differences in some brain regions. An FC difference in the cerebellar vermis IV/V was not observed between the PD-RBD and PD-nRBD groups, even in the presence of structural differences, which may boil down to this sample-size limitation. Second, the sex matching of the three groups may be suboptimal though the chi-square test among the three groups did not show a significant difference, but the differences between groups existed, especially in PD-RBD groups and other groups. Considering the subtle structural differences in the brain between males and females, the possibility of affecting the results cannot be ruled out [45]. Therefore, we should increase the sample size, enhance the PSG of all subjects, and match better the sex in future studies.

## 5. Conclusion

Higher GMV in the cerebellum may be linked with the abnormal motor behaviors during REM sleep in patients with PD-RBD, and lower GMV and FC in the posterior regions may indicate that PD-RBD correspond to more serious neurodegeneration, especially the visuospatial–executive function impairment and delayed memory impairment. These findings provided new insights to learn more about the complicated characteristics in patients with PD-RBD.

## Figures and Tables

**Table 1:** Demographic and clinical characteristics of all subjects.

Items	PD-RBD (*N* = 24)	PD-nRBD (N=26)	HC (*N* = 26)	*P* value
Age (years)	66.04 ± 5.73	62.96 ± 7.41	62.73 ± 6.33	0.15
Education (years)	11.00 ± 3.19	10.58 ± 2.80	11.62 ± 2.35	0.43
Sex (female/male)	4/20	10/16	12/14	0.07
MoCA	26.33 ± 1.52	26.46 ± 1.61	27.27 ± 1.67	0.08
MoCA-visuospatial skills/executive function	3.17 ± 1.05^a,b^	3.77 ± 0.77^b^	4.23 ± 0.65	<0.05
MoCA-naming	2.71 ± 0.55	2.85 ± 0.37	2.81 ± 0.40	0.66
MoCA-attention	5.79 ± 0.51	5.81 ± 0.57	5.69 ± 0.62	0.59
MoCA-language	2.83 ± 0.38	2.92 ± 0.27	2.96 ± 0.20	0.28
MoCA-abstraction	1.67 ± 0.57	1.69 ± 0.47	1.88 ± 0.33	0.20
MoCA-delayed memory	2.88 ± 1.36^b^	3.42 ± 0.90	3.85 ± 0.78	<0.05
MoCA-orientation	5.83 ± 0.38	5.96 ± 0.20	5.88 ± 0.33	0.33
HAMD	6.29 ± 2.85	6.38 ± 3.22	5.04 ± 1.69	0.13
RBDSQ	7.25 ± 1.48^a,b^	2.58 ± 1.39	1.81 ± 1.27	<0.05
Tonic chin EMG density scores	41.23 ± 22.77^a,b^	10.77 ± 6.10	9.14 ± 4.13	<0.05
Phasic chin EMG density scores	13.87 ± 5.67^a,b^	5.77 ± 3.54	6.54 ± 3.75	<0.05
Disease duration (years)	6.79 ± 3.58	5.69 ± 2.88	NA	0.24
UPDRS-IIIAR scores	29.88 ± 12.39	26.50 ± 10.34	NA	0.30
	19.63 ± 7.86	18.69 ± 8.03	NA	0.68
H&Y stage	2.50 ± 0.93	2.33 ± 0.86	NA	0.50
LEDD (mg/day)	607.96 ± 251.85	547.75 ± 190.86	NA	0.34

*UPDRS-III*, Unified PD Rating Scale-III; *H&Y stage*, Hoehn & Yahr stage; *MoCA*, Montreal Cognitive Assessment; *HAMD*, Hamilton Depression Rating Scale; *RBDSQ*, REM sleep behaviors disorder screening questionnaire; *AR*, akinetic/rigidity; *LEDD*, Levodopa equivalent daily dose; NA, not applicable. *P* < 0.05 was considered significant. *P* < 0.05, differs from the PD-nRBD group; ^*b*^*P* < 0.05, differs from the HC group. One-way analysis of variance analysis was applied in the analysis of age, MoCA, HAMD, RBDSQ, tonic chin EMG density scores, and phasic chin EMG density scores; Kruskal–Wallis test was applied in the analysis of education and MoCA domain scores; Chi-square was applied in the analysis of gender; two-sample *t*-test for disease duration, UPDRS-III, AR scores, H&Y stage, LEDD.

**Table 2:** One-way ANOVA of GMV and FC differences among PD-RBD, PD-nRBD, and HCs groups.

Brain regional	BA	Coordinates MNI			Clusters sizes	Peak *F* value
		*x*	*y*	*z*		
*GMV*						
Right SOG	18, 19	27	−93	21	87	14.42
Cerebellar vermis IV/V	—	3	−48	8	344	18.92
Left amygdala, extending to bilateral CN, bilateral putamen	36	−18	0	−15	838	31.83
Right angular	39	36	−66	51	368	16.99
Right insula	13	39	24	−3	60	12.33
right cerebellum lobule VI	—	27	−72	−18	124	10.28
Left MCG	6, 24	0	−6	45	93	15.79

*FC of right SOG*						
Left FG	37	−33	−75	−15	51	11.03
Left CS	17	−12	−90	0	237	13.03
Left SPG	7	−30	−60	51	48	9.02
Right SPG	7	36	−60	57	29	10.62
Right MCG	30	0	−15	48	23	10.46
Left cerebellum crus II	—	−21	−91	−33	32	12.55

The significance level of GMV was set at cluster-level corrected *P* < 0.05 with AlphaSim correction with voxel-level *P* value <0.001. *BA*, Brodmann's area; *MNI*, Montreal Neurological Institute; *SOG*, superior occipital gyrus; *CN*, caudate nucleus; *MCG*, middle cingulate gyrus; *FG*: fusiform gyrus; *LG*, lingual gyrus; *CS*, calcarine sulcus; *SPG*, superior parietal gyrus.

**Table 3:** Behavioral correlations with abnormal GMV in PD-RBD and PD-nRBD group.

	Right SOG		Cerebellar vermis IV/V		Left amygdala		Right angular		Right insula		Right cerebellum lobule VI		Left MCG	
	*r*	*P*	*r*	*P*	*r*	*P*	*r*	*P*	*r*	*P*	*r*	*P*	*r*	*P*
*PD-RBD group*														
MoCA	0.27	0.21	0.23	0.28	0.18	0.40	0.05	0.81	0.13	0.56	0.03	0.89	0.09	0.69
MoCA-visuospatial skills/executive function	0.29	0.89	−0.06	0.80	0.06	0.78	0.08	0.71	−0.04	0.84	−0.07	0.76	−0.19	0.38
MoCA-naming	−0.16	0.47	−0.07	0.74	−0.28	0.18	−0.17	0.44	0.12	0.58	−0.33	0.12	−0.04	0.87
MoCA-attention	0.08	0.71	−0.07	0.75	0.33	0.11	0.37	0.07	0.23	0.28	0.38	0.07	0.17	0.42
MoCA-language	0.45	0.03^*∗*^	−0.10	0.65	0.40	0.05	0.34	0.11	0.32	0.12	0.40	0.05	0.45	0.03
MoCA-abstraction	−0.31	0.14	−0.19	0.36	−0.16	0.47	−0.27	0.21	−0.21	0.32	−0.21	0.32	−0.00	0.99
MoCA-delayed memory	0.50	**0.01** ^*∗*^	−0.14	0.49	0.13	0.54	0.10	0.64	0.12	0.56	0.24	0.26	0.19	0.39
MoCA-orientation	−0.05	0.82	0.37	0.07	0.08	0.71	−0.16	0.45	0.05	0.82	0.18	0.41	0.10	0.65
Tonic chin EMG density scores	0.19	0.37	0.54	**0.01** ^*∗*^	0.16	0.45	0.16	0.45	0.11	0.63	0.15	0.49	−0.09	0.69
Phasic chin EMG density scores	0.10	0.64	−0.04	0.85	0.09	0.68	−0.05	0.83	0.11	0.60	0.06	0.80	−0.06	0.77
UPDRS-III	−0.44	**0.03** ^*∗*^	−0.14	0.52	−0.14	0.53	−0.34	0.10	−0.33	0.11	−0.27	0.21	−0.18	0.39

*PD-nRBD group*														
MoCA	0.12	0.55	−0.27	0.19	0.25	0.08	0.11	0.59	0.19	0.37	−0.09	0.68	0.13	0.52
MoCA-visuospatial skills/executive function	0.04	0.84	−0.05	0.80	0.13	0.37	0.24	0.25	0.32	0.12	0.27	0.18	−0.09	0.67
MoCA-naming	−0.14	0.49	−0.07	0.73	−0.05	0.76	−0.06	0.78	−0.09	0.68	−0.06	0.78	−0.07	0.73
MoCA-attention	0.05	0.82	−0.19	0.36	0.20	0.16	−0.44	**0.03** ^*∗*^	−0.20	0.33	−0.29	0.16	0.17	0.41
MoCA-language	−0.25	0.22	−0.04	0.85	0.22	0.12	0.00	1.00	0.14	0.51	0.00	1.00	−0.19	0.35
MoCA-abstraction	0.19	0.36	−0.13	0.52	−0.06	0.71	0.11	0.59	0.28	0.17	−0.17	0.42	−0.17	0.42
MoCA-delayed memory	0.05	0.80	0.03	0.90	0.17	0.25	0.34	0.09	0.18	0.39	−0.16	0.45	0.17	0.42
MoCA-orientation	0.12	0.56	0.04	0.85	0.15	0.30	0.01	0.95	0.33	0.10	0.33	0.10	0.09	0.65
Tonic chin EMG density scores	0.02	0.94	0.17	0.41	−0.10	0.48	−0.04	0.86	0.26	0.19	0.14	0.51	−0.36	0.07
Phasic chin EMG density scores	−0.26	0.19	0.15	0.45	−0.16	0.26	−0.15	0.47	0.08	0.70	0.19	0.36	−0.18	0.36
UPDRS-III	−0.12	0.58	0.42	**0.04** ^*∗*^	−0.16	0.28	−0.19	0.36	0.19	0.35	0.02	0.93	0.28	0.16

^*∗*^Statistical significance *P* < 0.05.

**Table 4:** Behavioral correlations with abnormal FC in PD-RBD and PD-nRBD group.

	Right SOG-left FG		Right SOG- left CS		Right SOG- left SPG		Right SOG-right SPG		Right SOG-right MCG		Right SOG-left cerebellum II crus II	
	*r*	*P*	*r*	*P*	*r*	*P*	*r*	*P*	*r*	*P*	*r*	*P*
*PD-RBD group*												
MoCA	0.01	0.98	−0.34	0.10	0.52	**0.01** ^*∗*^	−0.10	0.65	0.25	0.24	−0.27	0.20
MoCA-visuospatial skills/executive function	0.08	0.72	−0.13	0.56	0.66	**0.00** ^*∗*^	−0.04	0.84	0.24	0.26	−0.18	0.40
MoCA-naming	−0.29	0.17	0.13	0.54	−0.10	0.64	0.54	**0.01** ^*∗*^	0.22	0.31	−0.11	0.62
MoCA-attention	0.31	0.14	0.28	0.19	−0.39	0.06	−0.04	0.84	0.22	0.31	−0.27	0.21
MoCA-language	−0.29	0.17	−0.16	0.45	0.05	0.82	0.26	0.22	−0.03	0.88	0.17	0.43
MoCA-abstraction	0.11	0.60	−0.11	0.62	0.28	0.18	−0.17	0.42	−0.01	0.96	−0.20	0.35
MoCA-delayed memory	−0.14	0.53	0.41	**0.04** ^*∗*^	−0.23	0.28	0.26	0.22	0.00	0.99	0.06	0.77
MoCA- orientation	0.26	0.22	−0.23	0.29	−0.11	0.60	−0.18	0.41	0.00	0.99	−0.20	0.34
Tonic chin EMG density scores	−0.14	0.53	−0.10	0.64	−0.07	0.75	0.02	0.91	−0.11	0.62	0.15	0.49
Phasic chin EMG density scores	0.05	0.82	0.29	0.17	−0.14	0.52	−0.190.38	0.38	0.02	0.91	−0.03	0.89
UPDRS-III	0.10	0.65	−0.11	0.63	0.22	0.31	0.11	0.62	−0.35	0.10	−0.22	0.30

*PD-nRBD group*												
MoCA	−0.02	0.94	−0.11	0.58	0.09	0.68	−0.01	0.98	−0.13	0.52	0.16	0.43
MoCA-visuospatial skills/executive function	−0.18	0.37	−0.18	0.38	0.01	0.98	0.02	0.91	−0.32	0.11	0.30	0.13
MoCA-naming	0.11	0.58	−0.16	0.44	−0.04	0.84	−0.07	0.73	0.07	0.73	0.06	0.78
MoCA-attention	−0.24	0.24	−0.22	0.28	−0.05	0.80	−0.04	0.87	0.22	0.28	−0.47	**0.02** ^*∗*^
MoCA-language	−0.14	0.51	−0.14	0.51	0.27	0.18	−0.08	0.71	−0.21	0.30	0.02	0.93
MoCA-abstraction	0.09	0.67	0.07	0.75	−0.10	0.63	−0.01	0.96	−0.01	0.96	0.38	0.06
MoCA-delayed memory	0.03	0.88	0.09	0.67	0.05	0.81	−0.08	0.71	−0.04	0.87	0.20	0.32
MoCA- orientation	−0.25	0.21	−0.15	0.48	−0.25	0.21	−0.20	0.33	−0.31	0.13	0.33	0.10
Tonic chin EMG density scores	0.13	0.54	0.17	0.41	0.14	0.51	0.11	0.58	−0.06	0.77	0.31	0.13
Phasic chin EMG density scores	−0.25	0.22	0.03	0.90	0.02	0.92	−0.16	0.43	−0.19	0.36	0.37	0.06
UPDRS-III	−0.02	0.92	−0.05	0.83	−0.16	0.44	−0.07	0.72	−0.01	0.99	−0.46	**0.02** ^*∗*^

^*∗*^Statistical significance *P* < 0.05.

## Data Availability

The data used to support the findings of this study are available from the corresponding author upon request.
